# Metabolomic Study of 7-Ethyl-9-(*N*-methyl)aminomethyl-10-hydroxycamptothecin Derivative (NMe)—The Chemotherapeutic Drug Candidate Versus Irinotecan (IR) on a Mouse Model

**DOI:** 10.3390/metabo16030172

**Published:** 2026-03-05

**Authors:** Piotr Surynt, Beata Naumczuk, Magdalena Popławska, Magdalena Urbanowicz, Katarzyna Unrug-Bielawska, Magdalena Cybulska-Lubak, Zuzanna Sadowska-Markiewicz, Jerzy Sitkowski, Elżbieta Bednarek, Natalia Zeber-Lubecka, Lech Kozerski, Michał Mikula, Jerzy Ostrowski

**Affiliations:** 1 Department of Genetics, Maria Skłodowska-Curie National Research Institute of Oncology, Roentgena 5, 02-781 Warsaw, Poland; 2 Laboratory for Analysis of Bioactive Compounds, Institute of Organic Chemistry Polish Academy of Sciences, Kasprzaka 44/52, 01-224 Warsaw, Poland; 3 Falsified Medicines and Medical Devices Department, National Medicines Institute, Chełmska 30/34, 00-725 Warsaw, Poland; 4 Department of Gastroenterology, Hepatology and Clinical Oncology, Centre of Postgraduate Medical Education, Roentgena 5, 02-781 Warsaw, Poland

**Keywords:** irinotecan, NMe, metabolites, metabolomic study, ADME study, tissue distribution, renal excretion, comparative study, high-resolution mass spectrometry (HRMS), tandem mass spectrometry

## Abstract

Background: In this study, we aimed to compare metabolomic profiles, biodistribution, and detoxification patterns of the novel SN-38 derivative NMe with irinotecan (IR), and to identify NMe-specific metabolites to evaluate its preclinical pharmacokinetic advantages. Methods: In vivo ADME studies were conducted for NMe, a 9-aminomethyl SN-38 derivative, and IR following a single intraperitoneal dose of 40 mg/kg in mice. Additionally, ADMET properties were predicted using ADMETlab and SwissADME tools for comparison. Levels of NMe and irinotecan absorbed into plasma, distributed to tissues, and metabolized were monitored in liver, lung, spleen, kidney, and stool samples at 15, 30, and 60 min post-administration. Tissue extracts were analysed using high-performance liquid chromatography (HPLC), liquid chromatography–electrospray ionization quadrupole time-of-flight-tandem mass spectrometry (LC-ESI-QTOF-MS), and nuclear magnetic resonance (NMR) techniques after lyophilization and reconstitution. We compared the metabolomic profiles of irinotecan and NMe. Results: We identified and confirmed NMe-specific metabolites, including 9-CH_2_-S-cysteine conjugate, 9-CH_2_OH, and NMe-formyl. Notably, novel irinotecan metabolites (IR-OH and IR-ΔE) were detected in small amounts in kidney samples. In some cases, two literature-known photodegradation products of irinotecan were present. NMe was found to quickly metabolize with different distribution to tissues, significantly greater to kidney and liver. Two SN-38 glucuronides, SN-38G(α) and SN-38G(β), were detected corresponding to α- and β-anomers. Where it was possible, NMe, IR and SN-38 were quantified using external calibration curves. In IR group, controlled and prolonged release of SN-38 was confirmed in all samples, yet SN-38G was observed in minority only in plasma, kidney, or lungs. In NMe groups, great relative amounts of SN-38 and SN-38G were detected. Greater content of SN-38G in NMe group than in irinotecan is expected to contribute to modulation and alleviation of some side effects in irinotecan-involved therapies, such as gastrointestinal toxicities (GIT). Conclusions: NMe shows a distinct metabolic profile characterized by rapid biotransformation, higher systemic glucuronidation of SN-38, and formation of unique metabolites, suggesting a potentially wider therapeutic window and reduced toxicity compared with IR.

## 1. Introduction

**Figure 1:**
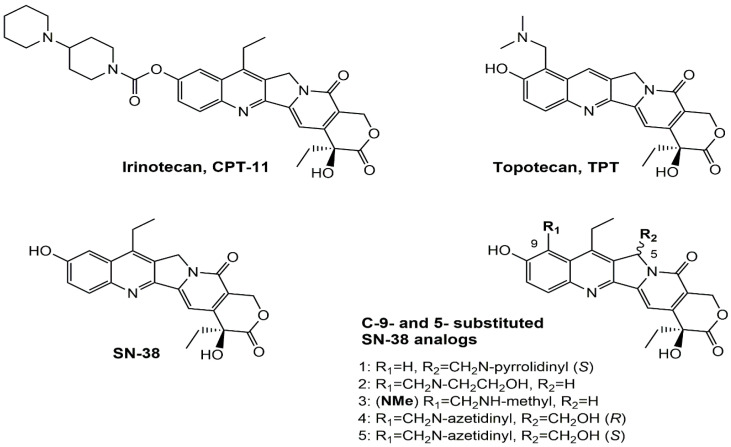
Structures of reference chemotherapeutic compounds (IR, TPT, and SN-38) and novel 5- and 9-functionalized SN-38 derivatives 1–5. NMe (3) was chosen for metabolomic studies.

Irinotecan (IR, also known as CPT-11, Figure 1) is a semi-synthetic compound derived from camptothecin (CPT) and is utilized for the treatment of colorectal cancer (CRC) [1,2,3,4] and other solid tumour malignancies [5,6,7,8]. The lactone form of 20-S camptothecin enantiomers binds to nicked DNA within the topoisomerase I cleavage complex. This interaction prevents religation of the nicked DNA strand, halts replication, and ultimately induces cell death [9,10,11]. Since the 1970s, numerous CPT derivatives have been synthesized and tested in clinical trials [12,13]; however, only irinotecan, topotecan, and two antibody-drug conjugates (fam-trastuzumab-deruxtecan and sacituzumab govitecan) have been approved for clinical use [14]. Also, many strategies for better delivery and efficacy have been proposed for irinotecan: liposomes [15,16,17], micelles nanocarriers, metal complexes, nanoferritin, and others [18,19,20,21,22,23,24]. Some of them successfully enabled to overcome limitations associated with camptothecin use: poor water solubility [25] and chemical pH-dependent instability [26]; however, the dose-related toxicity of irinotecan remained [27].

The new water-soluble SN-38 derivative, 7-ethyl-9-(N-methylamino)-9-hydroxyme-thyl-10-hydroxycamptothecin (NMe) [28], exhibits a unique mechanism involving spontaneous alkylation of aromatic DNA bases, resulting in highly effective topoisomerase I inhibition [9]. Consequently, in breast, leukaemia, colon, and lung cancer cell lines, the in vitro activity of similar compounds is several orders of magnitude greater than that of irinotecan used clinically as an API in Pfizer CAMPTO formulations [29]. Limited conversion of these derivatives to SN-38 suggests a potential reduction in therapy-related side effects.

IR and NMe were tested in vivo and in vitro in 3D cultures using seven developed colorectal cancer (CRC) patient-derived xenografts (PDX) [30]. In CRC PDX models, NMe showed comparable or enhanced therapeutic effects compared to irinotecan. In cells, NMe demonstrated similar to SN-38 tumour in vitro cytotoxic activity. Based on these preliminary findings, NMe may represent a promising breakthrough in chemotherapy.

In this work, we compared metabolite profiles, tissue biodistribution, and renal and fecal excretion of IR and NMe (20*-S* enantiomers) on immunodeficient, non-tumour-bearing mouse models, while also identifying novel metabolites and monitoring the chiral stability of the 20-*S* configuration in the most abundant fractions. Potential sex-related differences in drug metabolism were considered, as such variability may influence pharmacokinetics and therapeutic outcomes.

## 2. Materials and Methods

Irinotecan S enantiomer and SN-38 were purchased from Sigma Aldrich; irinotecan R enantiomer was purchased from Toronto Research Chemicals TRC; NMe derivative was synthesized as described previously [28]. CH_3_COOH acid was purchased from Pol-Aura; deuterated CH_3_CN and H_2_O were purchased from Eurisotop (Saint-Aubin, France); maleic acid was purchased from Sigma Aldrich (St. Gallen, Switzerland). CH_3_CN of LC–MS grade was purchased from Merck Millipore (LiChrosolv; Darmstadt, Germany) or Honeywell (Seelze, Germany), formic acid of LC-MS grade from Honeywell, and ultrapure H_2_O (18.2 MΩ.cm resistivity) with using Arium Comfort H_2_O-I-1-UV-T Ultrapure Water System from Sartorius (Goettingen, Germany) was used throughout.

**Figure A1:**
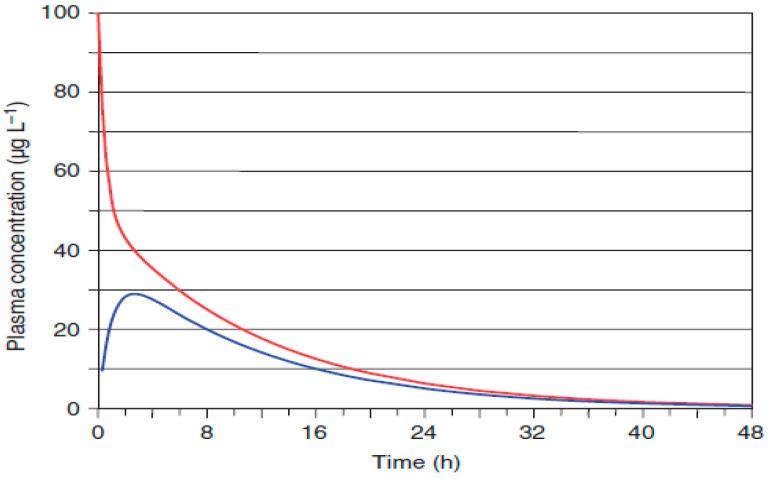
Absolute concentrations of drugs administered at the same dose by the intravascular (red) and extravascular (blue) route. Assuming constant and identical CL and identical, the AUC is 0.7, meaning that 70% of the administered dosed drug reaches the systemic circulation intact.

**Procedure for extraction and approach for metabolomic study**. Adult NSG/J mice (20 males and 20 females) were housed under SPF conditions with controlled temperature, humidity, and a 12 h light cycle. Animals were randomly assigned to treatment groups receiving a single intraperitoneal dose of NMe or irinotecan (40 mg/kg) or vehicle (0.9% NaCl). The choice of administration route is explained in Appendix A and Figure A1. Samples of plasma, liver, kidney, lung, spleen, and stool were collected at 15, 30, and 60 min post-dose and stored at −80 °C until analysis. All experimental procedures with laboratory animals were performed in accordance with the EU Directive 2010/63/EU and approved by the 2nd Local Ethics Committee for Animal Experimentation in Warsaw (WAW2/117/2018). Tissue samples were cut on dry ice and extracted under conditions ensuring lactone stability with acidified CH_3_CN/H_2_O 1:1, *v*/*v*, 1% CH_3_COOH, pH 3.2 using Diagenode Bioruptor Pico Picoruptor (Diagenode, Belgium) at 3 °C with 12 cycles of 60 s sonication and 30 s pause (60 s ON/30 s OFF). After centrifugation (10,000× *g*, 4 °C, 10 min), supernatants were collected, and the pellets were resonicated in an acidified CH_3_CN/H_2_O mixture and centrifuged, as previously. The combined supernatants were lyophilized and stored at −80 °C until nuclear magnetic resonance (NMR) analysis.

**NMR measurements**. Lyophilized samples were reconstituted in CD_3_CN/D_2_O (1:1 *v*/*v*) adjusted to pH 3.4, sonicated, and centrifuged. Supernatants were transferred to NMR tubes with TSPA-d4 as internal reference. The reconstituted solution’s pH was in range from 6.0 to 6.4. The reference compounds were dissolved and treated the same way. ^1^H NMR and 2D TOCSY spectra were acquired, and the spectra from studied groups were compared with the appropriate control group spectra. The cross peaks were tentatively assigned using available reference compounds (NMe, IR, SN-38, 9-CH_2_-OH, and SN-38-glucuronide). The same solutions were further subjected to high-performance liquid chromatography (HPLC), liquid chromatography coupled with quadrupole time-of-flight-tandem mass spectrometry (LC-QTOF-MS) analysis from which Rt, areas, and structural formulas of metabolites were obtained. The NMR spectra were recorded at 25 °C using a Varian VNMRS-500 spectrometer (Varian Inc., NMR systems, Palo Alto, CA, USA) operated at 499.8 MHz for ^1^H measurements. All experiments were run using the standard Varian software (VnmrJ version 3.1A software from Varian Inc., Palo Alto, USA). The spectrometer was equipped with an inverse ^1^H 5 mm Z-SPEC Nalorac IDG500-5HT probe (Nalorac Corp., Martinez, CA, USA) with an actively shielded z-gradient coil. The NMR spectra were referenced using sodium 3-trimethylsilyltetradeuteriopropionate (TSPA-d4) as chemical-shift internal reference. 2D TOCSY spectra were acquired using standard parameters (spectral width 8000 Hz, 32 scans, mixing time 30 ms).

**HPLC metabolomic analysis**. Chromatographic separation was performed on a Gemini 5 µm NX-C18 column (110 Å size, 250 × 4.6 mm, Phenomenex, Torrance, CA, USA) on Shimadzu system (described further in section LC-MS analyses) with mobile phases of A-aqueous 0.1% HCOOH and B-CH_3_CN at a flow rate of 1 mL/min using the following gradient: 10% CH_3_CN from 0 min, to 15% at 7 min, to 30% at 15 min, and to 50% at 20 min. Chromatograms were registered with UV detection at 260 nm wavelength.

**HPLC enantiomeric purity analysis**. For enantiomeric purity assessment, Waters e2695 HPLC system equipped with autosampler, quaternary pump, degasser, and UV detector was used. The output signal was monitored and processed using Empower3 software. The chromatographic column was Lux Cellulose-1 (150 × 4.6 mm, 5 µm, Phenomenex). The mobile phase was *n*-hexane:ethanol (46:51, *v*/*v*). The flow rate of the mobile phase was 0.9 mL/min. The column temperature was maintained at 30 °C, and the eluent was monitored at two wavelengths: 255 nm and 390 nm. The injection volume was 20 µL for standards and 30–50 µL for extracts depending on sample (pooled and single sample extracts). Solution of 20-(R) and (S)-enantiomers of irinotecan (5.5 and 4.0 mg/mL) was prepared in ethanol and diethyl-amine (250:0.7, *v*/*v*), which corresponds to 0.25% of modifier. Stock standard solution with mixture of 2.7 of 20-R and 2.0 mg/mL of 20-S IR enantiomers was prepared.

**LC-QTOF-MS metabolomic analyses**. Mass spectrometric analyses were performed using three different systems consisting of ultra-performance liquid chromatographs coupled with mass spectrometers (UPLC-MS). The first system, ACQUITY UPLC I-Class (Waters Inc., Milford, CT, USA), coupled with Synapt G2-S HDMS (Waters Inc.) mass spectrometer, was equipped with an electrospray ion source (ESI) and quadrupole time-of-flight (Q-TOF) type mass analyser. All chromatographic separations were carried out using the Acquity UPLC BEH Shield RP18 column (2.1 × 100 mm, 1.7 µm, Waters). The gradient elution started from 10% B after 7 min changed from 10% to 15% B in 8 min, then from 15% B to 30% B in 5 min, from 30% B to 50% B in 5 min, then from 50% to 100% B in 1 min and kept constant for 2 min, and finally returned to the initial conditions at a flow rate of 0.3 mL/min. Mobile phase A was water/formic acid (0.1%, *v*/*v*), and mobile phase B was CH_3_CN. The UV chromatograms were recorded at 260 nm and 365 nm.

The high-resolution mass spectra (HRMS) in ESI-positive mode were performed with the capillary voltage set to 2.5 kV. The desolvation gas flow was 600 L/h, and the temperature was 350 °C. The sampling cone voltage was 20V, and the source offset was set to 20 V and the source temperature to 120 °C. The data were obtained in a scan mode ranging from 100 to 1500 *m*/*z*. The leucine–enkephaline solution was used as the Lock-Spray reference material. The instrument was controlled, and recorded data were processed using the MassLynx V4.2 software package (Waters Inc., Milford, USA).

To properly identify HPLC-UV peaks, chromatographic separations in two other LC-QTOF-MS systems, i.e., Ultimate 3000 system from Dionex (Thermo Fisher Scientific, Waltham, MA, USA), coupled with a high-resolution micrOTOF-QII hybrid mass spectrometer with a TOF analyzer (Bruker Daltonik, Bremen, Germany) and chromatograph system with Q-TOF mass spectrometer LCMS-9050-Q-TOF (Shimadzu Corporation, Kyoto, Japan), were achieved using the same column and conditions as used in HPLC metabolomic analysis. To reduce eluate flow rate, post-column binary fixed flow splitter with split ratio 10:1 was added before the ESI source entrance. Analyses were conducted in positive ionization mode, and a calibration segment with a sodium formate solution was included in each sample run to ensure high mass accuracy.

MS settings (Ultimate 3000-micrOTOF-QII-MS) were as follows: the capillary voltage was set at 4.5 kV, end plate offset at −500 V, dry gas flow rate at 8.0 L/min, and dry heater at 190 °C. Data acquisition was performed in full scan mode over an *m*/*z* range of 50–1500, and the collision energy was linearly ramped based on the *m*/*z* in the fragmentation experiments. For *m*/*z* values between 200 and 400, the voltage increased linearly from 20 to 25 eV, while for range 400 and 800 *m*/*z*, it increased from 25 to 35 eV.

MS settings (LCMS-9050-Q-TOF) were as follows: nebulizing gas flow rate 3 L/min, drying gas flow rate 10.0 L/min, heating gas flow rate 10.0 L/min, interface temperature 300 °C, desolvation line temperature 250 °C, heat block 400 °C, interface voltage 4.0 kV, and full scan mode (from *m/z* 50 to 900) was chosen. Data-dependent analysis algorithm with 4 MS/MS events was selected. For two events, the collision energy (CE) was fixed at 30 V with a CE spread of ±10 V, and for the next two events, CE was set at 40 V with a spread of ±15 V. Additionally, a priority ion list with *m*/*z* values of potential metabolites was introduced into the acquisition method.

**Statistical evaluation**. Differences in metabolite relative amounts between study groups were assessed using the Mann–Whitney U-test. Mann–U Whitney and related statistical tests (Wilk–Shapiro and FDR tests) were performed with Graphpad prism 9.0 software (Dotmatics). The possible false discovery rate test (FDR) was assessed, and none were detected.

## 3. Results

We conducted a comprehensive metabolomic analysis of NMe and IR in murine plasma and tissues (liver, kidney, spleen, lungs, and stool), including metabolite identification (LC-QTOF-MS), profiling, and, where feasible, quantification using calibrated LC-QTOF-MS methods. Samples were processed under conditions ensuring lactone stability (pH < 4) and pooled where necessary to obtain sufficient material.

### 3.1. Metabolomic Profiles of NMe and IR

#### 3.1.1. Metabolite Identification

The identified compounds, including NMe, irinotecan, and their metabolites, detected in the extracted samples are summarized in Table 1 and Table 2. Structural formulas were confirmed by high-resolution mass spectrometry based on the observed *m*/*z* values.

**Figure 2:**
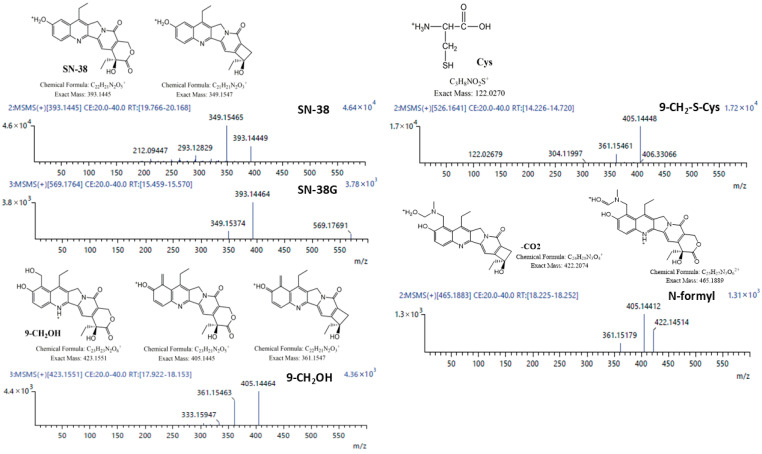
Product ion spectra with corresponding ion structures for all identified metabolites of NMe.

Tandem mass spectra for identified NMe metabolites supporting designated structures are presented below (Figure 2). Structural elucidation of the metabolites was performed using NMR spectroscopy whenever feasible.

The only metabolites common to both tested active compounds were SN-38 and SN-38G (Figure 2, Table 1 and Table 2). However, the origin of SN-38 differs for these two molecules: in irinotecan, it is generated by the removal of the substituent at the C-10 hydroxyl group, whereas in NMe, it arises via a retro-Mannich reaction. In our in vitro studies, NMe incubated in buffer at pH 6.5 exhibited a half-life of approximately 1000 h and primarily yielded the alcohol (9-CH_2_OH) as the main hydrolysis product, with only trace amounts of SN-38 formed via the retro-Mannich pathway.

#### 3.1.2. Tissue Distribution and Kinetics

**Figure 3:**
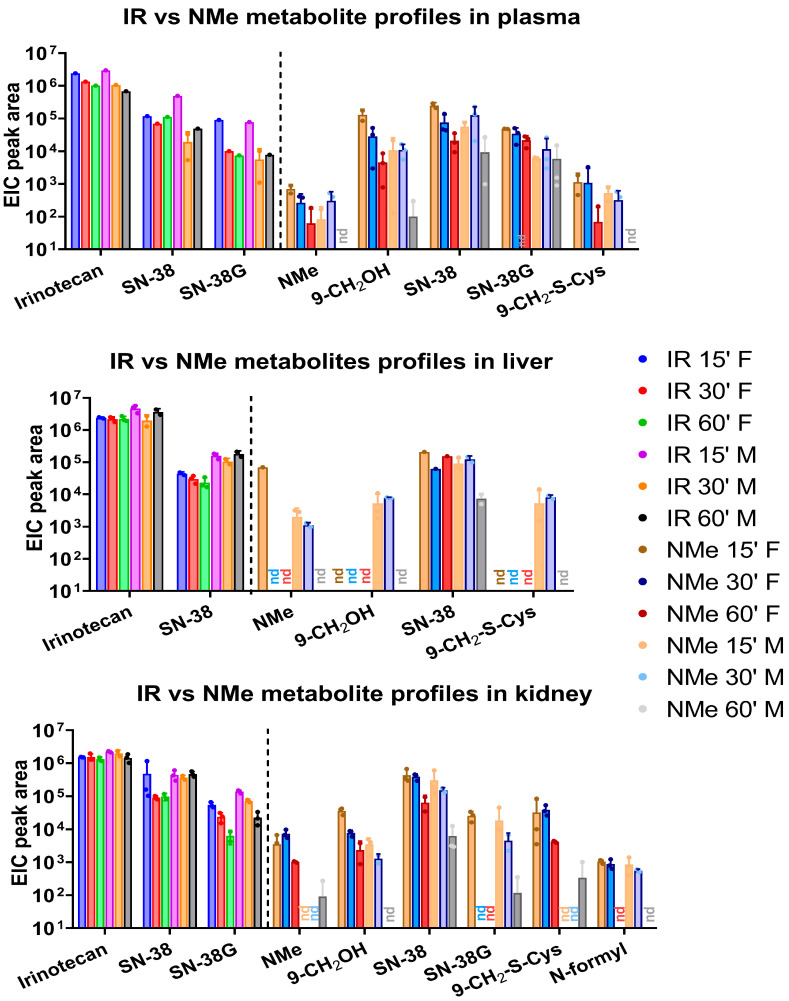
Metabolite profiles in NMe and IR groups in different compartments: plasma, kidney, and liver after 15, 30, and 60′ of administration with mean extracted ion chromatographic (EIC) peak area (relative amounts) and with separated genders; nd- not detected.

**Figure 4:**
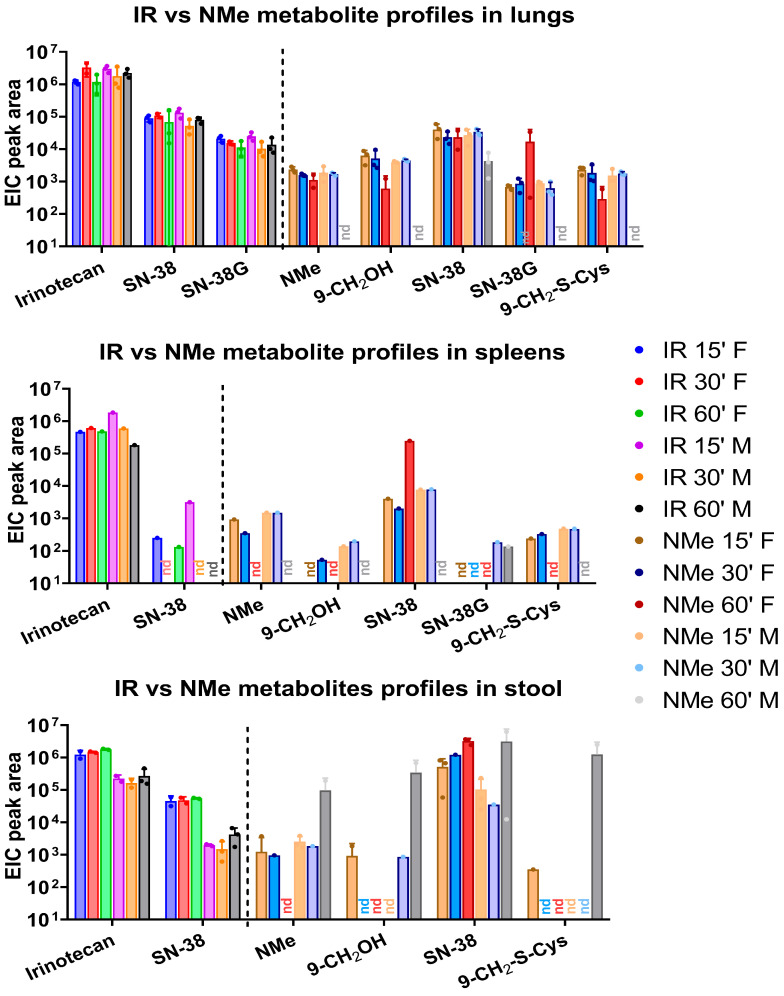
Metabolite profiles in NMe and IR groups in different compartments: spleens, lungs, and stool after 15, 30, and 60 min of administration with mean EIC peak area (relative amounts) and with separated genders; nd-not detected.

**Figure 5:**
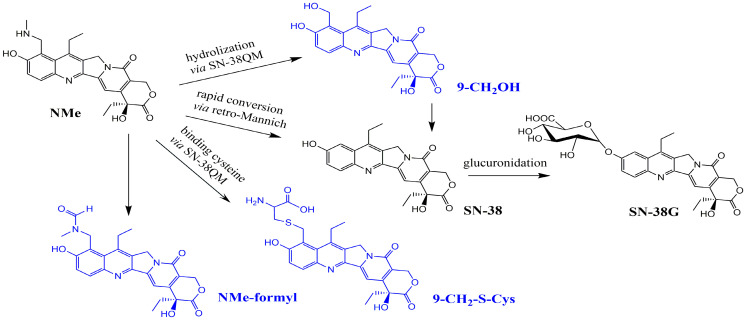
NMe metabolic pathway in mice determined in this study; metabolites specific to NMe were marked in blue.

**Figure 6:**
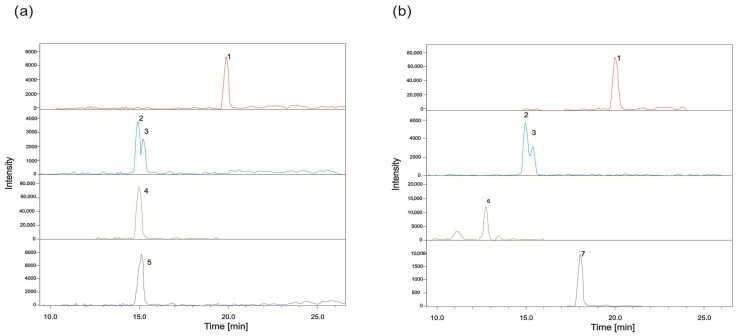
Extracted ion chromatograms of active compounds and their metabolites recorded in positive ion mode on the example of samples (**a**) 3F15-IR liver; (**b**) 9F15-NMe plasma: 1—SN-38, 2—SN-38G-α, 3—SN-38G-β, 4—IR, 5—PDP2, 6—NMe, and 7—9-CH_2_OH.

**Figure A3:**
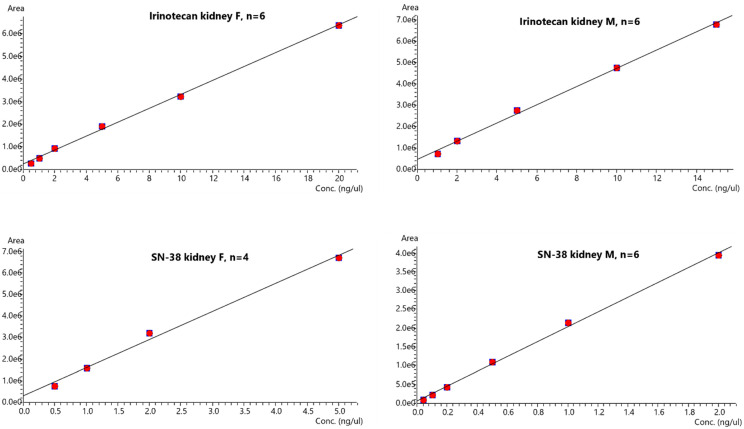
Calibration curves for IR and SN-38 used for kidney extract samples (F/M analysed on different dates).

**Figure A4:**
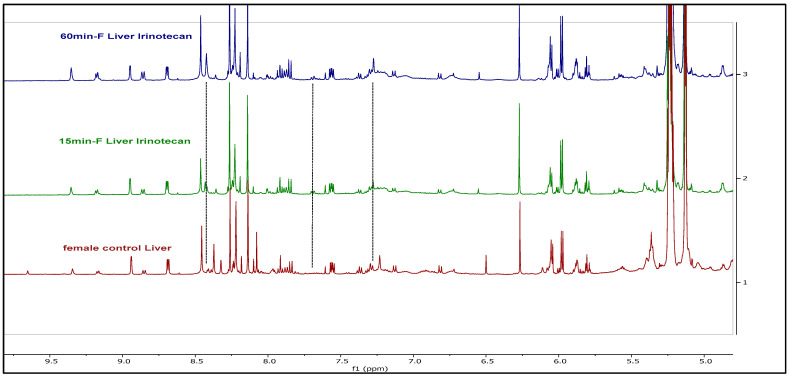
Comparison of ^1^H spectra of extracts from liver samples for irinotecan and different timepoints. Some peaks at 7.6, 8.1, and 8.35 ppm have been marked and identified as camptothecins.

**Figure A5:**
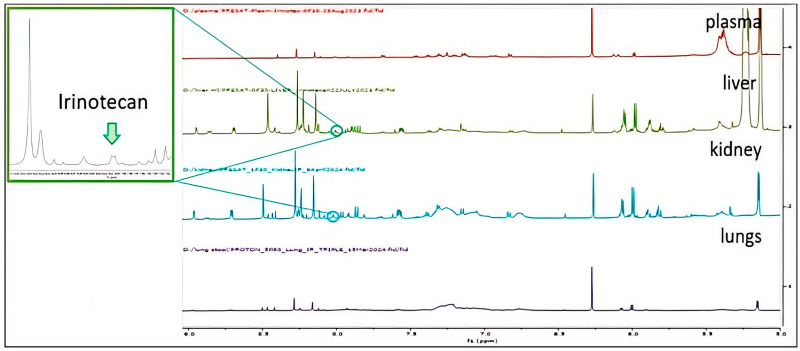
^1^H NMR spectra of aromatic region for extracts in different tissue samples or plasma, after irinotecan administration; doublet at ~8.01 ppm has been marked as diagnostic for irinotecan.

Analyses revealed consistently high concentrations of SN-38 across all examined tissues, accompanied by only minor amounts of NMe shortly after administration (Figure 3 and Figure 4). In contrast, the irinotecan-treated group exhibited the opposite trend: the parent compound underwent slow metabolic conversion, yielding only low SN-38 levels. In both groups, substantial amounts of SN-38G were detected in plasma, lungs, and kidneys. Besides the primary NMe metabolites, notable quantities of 9-CH_2_OH and its 9-CH_2_-S-cysteine conjugate were also identified. A schematic overview of the proposed NMe metabolic pathway in the murine model is presented in Figure 5. The alcohol and conjugate are likely formed in vivo through the hyper-reactive quinone, SN-38QM intermediate (Figure 6). Where feasible, active compounds and the main metabolite SN-38 were quantified (Figure A3, Figure A4 and Figure A5; Table A3, Table A4, Table A5 and Table A6). Due to insufficient integrals for NMe and other metabolites, quantification in the NMe group was not performed, except for SN-38 in selected samples (Table A5). Comparison of SN-38 levels between the two groups revealed 2–6-fold higher concentrations in plasma and kidney in NMe group (Table A6). The highest relative levels of SN-38 were observed in liver and kidney extracts in IR group. Although more sensitive analytical methods such as LC-MS/MS in multiple reaction monitoring (MRM) mode exist, they require prior knowledge of target transitions (e.g., for SN-38: *m*/*z* 393.1 → 349.1) and are therefore unsuitable for broad metabolite screening.

The data presented in Figure 3 and Figure 4 allow for several important comparisons between the two groups studied. In the IR-treated group, SN-38G was detected only in plasma, kidney, and lungs, with extracted ion chromatogram (EIC) peak areas of approximately 10^4^. In contrast, in NMe group, SN-38G was detected in all examined tissues except stool and liver, with EIC peak areas ranging from 10^2^ in spleen and lungs to as high as 10^4^ in plasma.

The metabolite 9-CH_2_-Cys was detected across all analysed tissues, with relative abundances ranging from 10^2^–10^5^ in the kidney and reaching up to 10^6^ in stool. However, data from stool extracts are less reliable for short-term analyses, as the timing of metabolite excretion into pre-formed stool within this matrix remains uncertain.

These findings indicate a rapid and predominant elimination pathway and suggest that this metabolite is formed through conjugation with free cysteine either in plasma and extracellular fluids followed by distribution to tissues or directly within specific tissues. Although free cysteine is synthesized and present in the liver, it effectively absorbs and subsequently transports to other organs, including the brain and kidneys. Cysteine conjugation in metabolite pathways is well-known reactive oxidative species inactivating and scavenging mechanism [32,33,34]. Dysregulation of cysteine, rather than the presence of its conjugates, is the primary concern.

The content of NMe and its primary hydrolysis product (9-CH_2_OH) in plasma extracts decreased significantly during storage at −80 °C over a 6-month period, in contrast to stable levels of other metabolites (SN-38, SN-38G, Table A5). These results indicate the comparatively low stability of NMe and 9-CH_2_OH in biological matrix under long-term storage conditions. A similar phenomenon has been reported for pancuronium [35], an aminosteroid anaesthetic, which degrades in aging tissue samples and in blood stored at −20 °C for more than 7 months, with approximately 20% reduction in analyte concentration after 1 year. The degradation kinetics of pancuronium indicate that its stability is strongly influenced by the biological matrix (liver, kidney, and plasma).

Representative EIC chromatograms from plasma (NMe group) and liver (IR group) are shown in Figure 6, illustrating tissue-specific detection of parent compounds and metabolites. The assignment of SN-38 glucuronide epimers was based on literature reports, indicating α-epimer predominance in biological systems; however, both epimeric forms can coexist in tissues. Consistent with this, lung and kidney extracts exhibited two signals with identical molecular mass and similar retention times (Figure 6, peaks 2 and 3), corresponding to the α and β epimers of SN-38G.

#### 3.1.3. Systemic Glucuronidation Index (SGI)

The data presented in Table 3 indicate that the systemic glucuronidation index (SGI) is roughly an order of magnitude higher in the NMe-treated group compared to the IR-treated group and increases over time, whereas it remains relatively constant for irinotecan. This finding suggests that SN-38 detoxification is more efficient in the NMe group, while in the IR group, the system remains exposed to toxic SN-38 for a longer duration, which is associated with diarrhoea.

**Figure 7:**
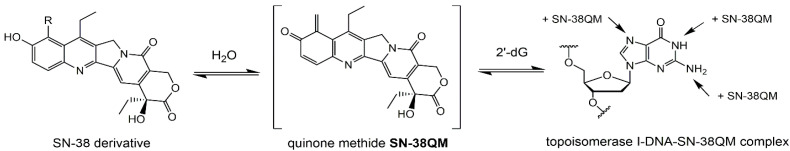
The intermediate o-methylene quinone alkylates the nitrogen bases in a biological target (nick in the DNA strand), leading to apoptosis of cancer cells.

SN-38QM (the quinone methide from SN-38) is considered both a fragmentation ion and a highly reactive intermediate state in 9-CH_2_OH, SN-38, and 9-CH_2_-S-Cys formation. This form cannot be isolated or quantified directly; however, in our previous work [28], its complexed form with DNA and topoisomerase in vitro was observed and registered in NMR spectra (Figure 7).

#### 3.1.4. Sex-Related Diversity

**Figure 8:**
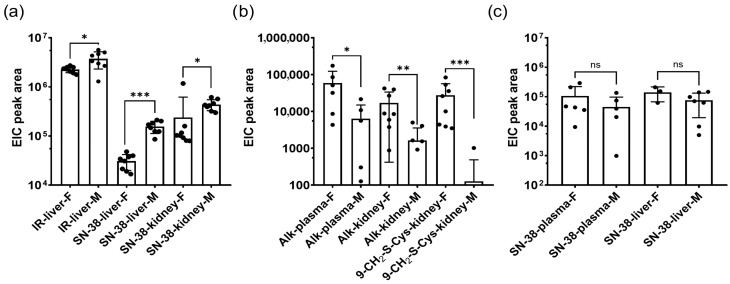
Mean EIC peak areas for drugs and metabolites and sex-related differences: (**a**) Some significant differences in IR group with higher values for males, (**b**) some significant differences in NMe group with higher values for females, and (**c**) insignificant differences for SN-38 in NMe group (in contrast to a IR group). Statistical significance between groups related to animal sex was assessed using Mann–U Whitney tests (* *p* < 0.05). Alk notes here: 9-CH_2_OH. ns = not significant; * *p* < 0.05; ** *p* < 0.01; *** *p* < 0.001.

Because drug metabolism and distribution can differ between sexes, we analysed sex-related differences in metabolite levels. Figure 8 illustrates several notable differences between male and female mice. In liver extracts from the IR group, concentrations of IR and SN-38 remained relatively stable. In contrast, at the corresponding timepoints in the NMe groups, higher levels of SN-38 were observed in females and comparable levels in males, with a decreasing trend over time and only trace amounts of NMe detected. This decline resulted in NMe and 9-CH_2_OH levels falling below the detection limit. In plasma, a clear downward trend was observed for both IR and NMe, as expected; however, for NMe, it is more significant. In male liver and female kidney samples from the NMe group, a transient increase at 30 min followed by a decrease at 60 min was noted, likely reflecting distribution and excretion processes. Conversely, in the IR-treated group, persistent levels of IR and its metabolites were detected in the liver, lungs, and stool, indicating a prolonged release of SN-38. In spleen samples from the NMe group, SN-38 was the most abundant metabolite, with similar levels at 15 and 30 min and either elevated (in females) or reduced (in males) levels at 60 min. In NMe group, significantly higher amounts for most metabolites were found in females (Alk;9-CH_2_OH: plasma- *p* = 0.0411, -kidney, *p* = 0.0070, -liver, *p* = 0.0079, 9-CH_2-_S-Cys: kidney, *p* = 0.0002). These findings should be interpreted with caution due to the small sample size and limited statistical power. At early timepoints, NMe and other metabolites were also detectable in spleen tissue.

NMe, IR, and their metabolites were identified in extracts by NMR techniques whenever feasible. In the complex spectra obtained from the studied samples, only the aromatic proton spin system proved sufficiently characteristic to allow reliable diagnosis and identification. Accordingly, the identification process was performed by comparing the spectra of reference compounds and controls with those obtained from the tested samples. IR was assigned on ^1^H 1D NMR spectra of liver or kidney extracts (Figure A4 and Figure A5). Some proton signals in liver extracts in IR group were increasing over time: ~7.62, 8.02, and 8.23, from which 8.02 ppm is diagnostic for irinotecan and enabled its identification (Figure A5). The singlet at 7.62 ppm appears in both IR and NMe standard solutions and is therefore not diagnostic. Despite that successful identification, 1D spectra were not suitable for quantification. The adopted NMR identification methodology for TOCSY spectra is illustrated with examples in Figure A7, Figure A8, Figure A9 and Figure A10). Specifically, ^1^H-^1^H TOCSY spectra of tested extracts samples were compared with appropriate reference compounds and samples from control group. The ^1^H NMR signals appear along the diagonal, and those belonging to NMe are distinguished by marked cross peaks absent in controls. In the TOCSY NMR spectrum of the β-D-SN-38 glucuronide standard (Figure A7), the β form predominated (97%); however, approximately 3% of α form was also assigned.

**Figure A6:**
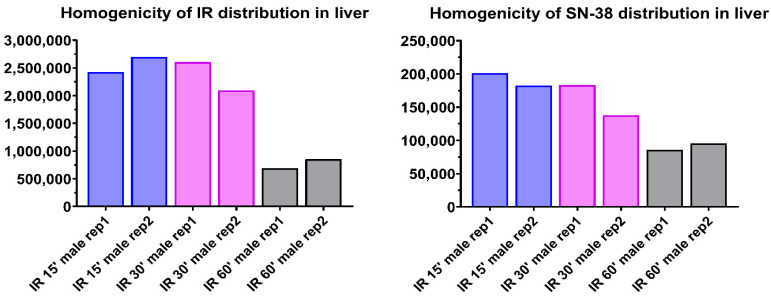
IR and SN-38 EIC peak areas for two independent liver samples extractions: 29M15 (blue), 26M30 (purple), and 30M60 (black).

**Figure A7:**
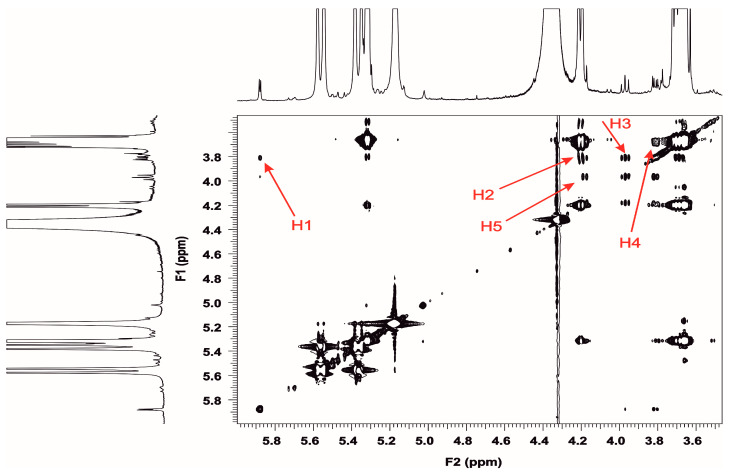
TOCSY spectrum of β-D-SN-38 glucuronide standard. The arrows in a figure show the glucose protons, which give rise to cross peaks in an α form. The large cross peaks belong to β form.

**Figure A8:**
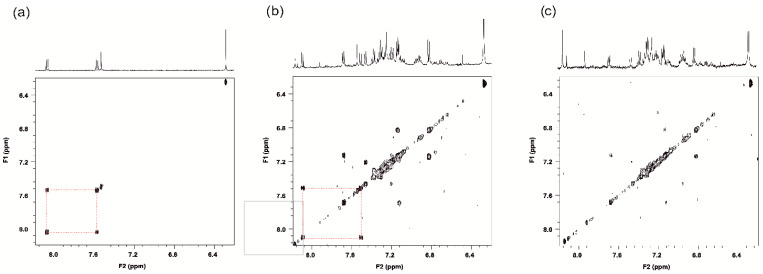
TOCSY and ^1^H NMR projection spectra zoomed into aromatic region of (**a**) 9-CH_2_OH standard, (**b**) plasma extract 9F15 NMe (symbols denote the mouse number, gender, and time in minutes of sacrifice after drug administration), and (**c**) plasma extract from control group. The red squares indicate the cross peaks, not found in control extract in TOCSY; doublets in ^1^H NMR denote the H12 and H11 protons of 9-CH_2_OH.

**Figure A9:**
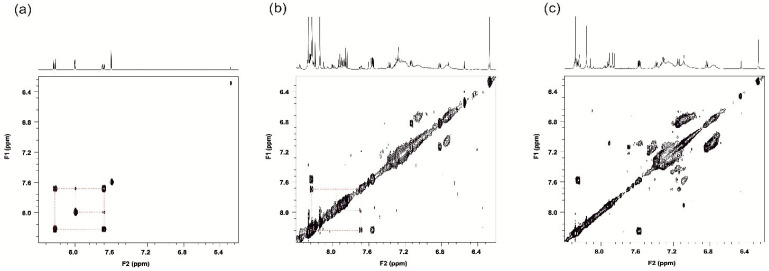
TOCSY and ^1^H NMR projection spectra of aromatic region of (**a**) IR standard, (**b**) extract 5F60 liver IR, and (**c**) liver extract of control group. The red squares indicate the cross peaks, not found in control extract in TOCSY; multiplets in ^1^H NMR denote the H12, H11, and H9 protons of IR.

To assess LC-QTOF-MS method’s repeatability, the plasma sample was reanalyzed after 6 months stored at −80 °C (12F60, NMe), and liver samples from males of the same animal were extracted and analyzed in two independent experiments (duplicates: liver 29M15, 26M30, and 30M60-NMe). Plasma extract results demonstrated good repeatability (Table A7) even after long-term storage, while replicated liver samples from the same animal showed high homogeneity and method’s precision (Figure A6).

### 3.2. Enantiomeric Purity Control of IR in Biological Matrices

**Figure A10:**
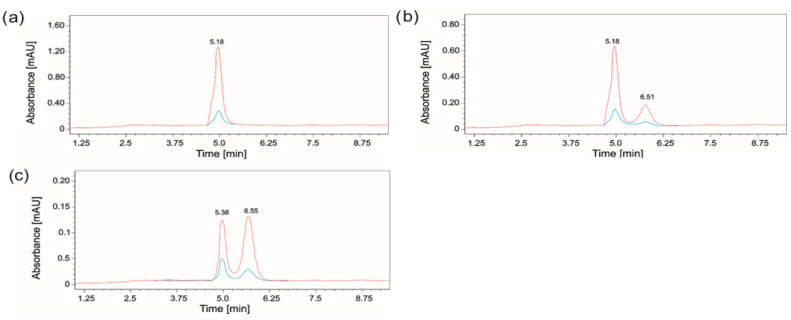
Chromatograms of IR enantiomers on chiral column with detection wavelengths at 255 nm (red) and 390 nm (blue) for (**a**) reference of R-IR, (**b**) R-IR and S-IR standards (3:1) co-injection, and (**c**) isolated fraction of IR from pooled liver males IR extract.

Compounds from the camptothecin family naturally predominantly exist in the (20S)-enantiomeric form, which exhibits biological activity and is consequently employed in clinical practice. Some HPLC methods with chiral stationary phase columns have been developed and validated in order to detect and quantify R and S-IR [36] and R/S-SN-38. To test whether the racemization process could occur in vivo, we co-injected pure (20*S*)-IR and (20*R*)-IR enantiomers and separated them on a column with a chiral stationary phase of tris(3,5-dimethylphenylcarbamate) cellulose (Figure A10a,b) according to validated method by [36], achieving satisfactory separation of the S- and R-enantiomers from pooled male liver extracts, with almost equal areas of (20*S*)-IR and (20*R*)-IR (Figure A10c). The R-IR enantiomer was not detected in freshly extracted lung or faecal samples. This IR enantiomerization at C20 is not observed in matrix-free biological solvents. Verification of the potential racemization process of NMe could not be performed due to its rapid biotransformation and too low levels, even for pooled liver extract samples.

To our knowledge, racemization of irinotecan at C20 has not been reported in the literature; meanwhile, most metabolomic studies do not explicitly consider the chirality or enantiomeric integrity of xenobiotics and metabolites. Therefore, the observation of an apparent racemate in biological extracts is unexpected and cannot be readily attributed to a known in vivo process. Importantly, this mixture of enantiomers may arise artificially during sample processing and analysis and may not reflect true biological racemization. Specifically, irinotecan and related camptothecins undergo pH-dependent lactone ring opening at pH > 6 in plasma and tissues, forming carboxylates with an sp^2^-hybridized carbonyl system that is planar, electronically distinct from the closed lactone, and prone to racemization. Extraction under acidic conditions (pH < 4) allowed complete relactonization, whereas repeated opening and closing cycles during extraction, lyophilization, or reconstitution may have facilitated stereochemical mixing. Furthermore, TOCSY analyses performed in D_2_O/CH_3_CN mixtures (pH ~5.5–6) could potentially promote lactone–carboxylate equilibrium under some conditions, such as long acquisitions. Taken together, these results suggest that the observed (20*R*), (20*S*) IR mixture in liver extracts likely reflects method-dependent enantiomerization via ring-opening and relactonization rather than intrinsic metabolic racemization. This finding highlights the importance of chirally aware analytical strategies in metabolomics, particularly for stereochemically labile drug molecules, and underscores the need for careful sample preparation and pH control when interpreting enantiomeric purity in biological matrices. Our findings encourage the monitoring of enantiomeric purity, especially in the context of some burdensome therapeutic regimens for patients, such as irinotecan.

## 4. Discussion

This study analysed the relative and absolute concentrations of irinotecan (IR) and NMe in plasma, liver, lung, spleen, kidney, and stool samples collected at 15, 30, and 60 min following a single intraperitoneal administration (40 mg/kg). Metabolite structures were characterized using HPLC, LC-QTOF-MS, ^1^H NMR, and 2D NMR TOCSY techniques. While both IR and NMe were primarily metabolized to SN-38 and SN-38G, we identified additional metabolites specific to NMe: 9-CH_2_-S-Cys conjugate, 9-CH_2_OH, NMe-formyl, and others to IR: novel derivatives IR-OH and IR-ΔE (Table 2 and Table 3; the latter detected exclusively in kidney samples). NMe in plasma was rapidly metabolized or transported after administration; higher relative concentrations were observed in the kidney and lungs compared to plasma (Figure 2 and Figure 3). The main metabolite was SN-38, but relative amounts of α- and β-SN-38G, corresponding to α- and β-glucose anomers, in NMe group were greater than in IR. Because SN-38G levels do not reflect antitumour efficacy but rather are the sole indicator of SN-38 detoxification, the higher SN-38G levels observed in the NMe group may be interpreted solely in the context of metabolic processing and potential tolerance and not as a measure of therapeutic activity.

In IR group, SN-38 has been of prolonged release and constant levels in all compartments, though SN-38G more differentiated with decreasing trends in time. Two literature-known irinotecan photodegradation products [31] were also identified. NPC was not found, while trace amounts of APC were found in liver extracts. Irinotecan photodegradation and hydrolyzation products (IR-OH, PDP2, and PDP5 were found in most extracts, except for stool and spleen, with relative amounts close to SN-38 (10^4^–10^5^ EIC peak area, Table 2). Additionally, an irinotecan degradation product lacking the E ring (IR-ΔE) was identified in kidney extracts, with no analogous product observed for NMe.

Metabolism of irinotecan involves many enzymes, including carboxylesterases (CES), butyrylcholinesterase (BES), uridine diphosphate glucuronosyltransferases (UGT), cytochrome P450 enzymes and β-glucuronidases. Irinotecan is hydrolysed into the active but highly toxic metabolite SN-38 by CES1, CES2, and BES. In turn, hepatic glucuronidases, UGT1A isoenzymes, convert SN-38 into inactive SN-38G [25,26,37,38,39,40,41], which is then excreted by the bile to intestinal tract, where it can be reconverted into SN-38 or be eliminated predominantly in faeces. Intrahepatic cytochrome P450 enzymes metabolize irinotecan into inactive metabolites APC and NPC. Furthermore, APC can be converted in small extent to SN-38 by CES in the liver. In hepatic microsomes, IR oxidation product with the loss of two hydrogen atoms from the piperidine ring was also reported [42]. NMe biotransformation to SN-38 likely occurs via a retro-Mannich reaction, with aminoalkyl moiety loss plausibly explained by structure-dependent iminium-mediated cleavage (pH- or enzyme-driven). Formation of an iminium ion during the retro-Mannich process leads to deamination. This highly reactive intermediate can undergo hydrolysis back to an aldehyde and an amine. A key step in described deamination process involves the loss of the amine moiety (C9 substituent). Some CYP enzymes can also promote mentioned ring deamination [43,44].

Sex-related differences were observed, with females exhibiting higher levels of key NMe metabolites in plasma, kidney, and liver. For instance, irinotecan exhibited significant sex- and time-of-day-dependent toxicity and pharmacokinetic patterns, with females showing more pronounced circadian disruption and differential expression of detoxifying enzymes (e.g., Ugt1a1 and Abcc2) [45,46]. Furthermore, rodent studies have documented sex-specific hepatic CYP450 expression profiles, which can critically influence drug clearance [47,48]. Although our sample size was small, these findings support the hypothesis that sex influences NMe metabolism and distribution. Future studies should investigate enzymatic and transporter expression differences to elucidate mechanisms and evaluate potential clinical implications.

Plasma SN-38G to SN-38 ratio is used as a surrogate marker of a patient’s ability to detoxify SN-38 through glucuronidation [27]. Low SN-38G/SN-38 (or to other metabolites) ratio suggests reduced glucuronidation capacity, higher exposure to SN-38 with an increasing risk of both camptothecin-induced diarrhoea and neutropenia. While a low ratio (IR group, Table 3) typically implies poor glucuronidation and systemic toxicity, a high ratio (NMe group, Table 3) does not preclude GI toxicity due to the role of microbial β-glucuronidase in regenerating SN-38 in the gut. Low ratios in patients’ blood, especially those with UGT1A1 polymorphisms, were significantly associated with higher rates of irinotecan-induced diarrhoea [49,50]. Understanding this ratio can guide personalized chemotherapy, minimize toxicity, and improve outcomes.

### Limitations and Further Directions

NMe is a promising drug candidate, which may serve as an effective alternative to irinotecan. NMe demonstrates greater water solubility than IR, high anticancer efficacy in CRC PDX models, and rapid metabolism and distribution in mice. Within 15 min after NMe administration, substantial amounts of SN-38, 9-CH_2_OH, and 9-CH_2_-S-Cys were detected, along with even higher levels of SN-38G compared to those observed after irinotecan administration. The question remains whether unique NMe metabolites, such as 9-CH_2_-S-Cys, have cytotoxic potential. Unfortunately, due to insufficient isolated metabolite amounts, we have not conducted investigations within this topic. Possible NAC and GSH conjugates were also searched, but none were found; however, this was a single-dose study, and those should not be excluded in repeat-dose administration. The increased formation of SN-38G suggests potentially reduced toxicity with NMe treatment. In contrast, irinotecan administration did not result in detectable levels of SN-38G in the liver, or its concentration remained below the limit of detection. This distinct metabolic profile and kinetics highlight a considerably wider therapeutic window for NMe.

In terms of tissue distribution, NMe showed a pattern similar to topotecan, with the highest relative concentrations in the kidney, followed by the lung, and the lowest in the plasma [51]. Autoradiographic studies of camptothecin analogues administered by different routes demonstrated consistent organ distribution patterns, including accumulation in the kidney, even when the overall plasma profiles differ [51]. However, a full comparative analysis of biodistribution between NME and TPT requires matched experimental conditions.

Mechanistically, however, NMe more closely resembles irinotecan, as its in vivo activity is partially mediated through SN-38. Importantly, NMe may interact with topoisomerase I both directly and via SN-38 (and other metabolites such as 9-CH_2_OH). In comparison, TPT in vivo undergoes minimal metabolism and acts directly on the topoisomerase I/DNA complex.

Given the differences in CES and UGT1 activities between humans and mice, more comprehensive preclinical toxicity studies are warranted. Future investigations should focus on metabolite identification and profiling in tumour samples, as well as the safety of NMe administration schemes, particularly in relation to the formation of cysteine, and possible NAC and GSH S-conjugates with plasma-free cysteine level monitoring.

## 5. Conclusions

NMe demonstrates a markedly different metabolic profile compared to irinotecan. It undergoes rapid biotransformation and preferential distribution to the kidney, accompanied by significantly higher systemic glucuronidation of SN-38. This enhanced detoxification suggests a potentially wider therapeutic window and reduced risk of irinotecan-associated toxicities, such as gastrointestinal side effects. The identification of unique NMe metabolites (9-CH_2_OH, 9-CH_2_-S-cysteine, and NMe-formyl) further highlights its distinct pharmacological behavior. Collectively, these findings support NMe as a promising chemotherapeutic candidate, warranting further preclinical evaluation.

## Figures and Tables

**Table 1:** Structures of NMe and metabolites detected and identified by high-resolution mass spectrometry (HRMS) and MS/MS fragmentation experiments in extracted biological samples.

Structure, Abbreviation, and |Formula	HRMS for [M + H]^+^ Calculated	HRMS Found (Error [ppm])	MS/MS Product Ion Masses
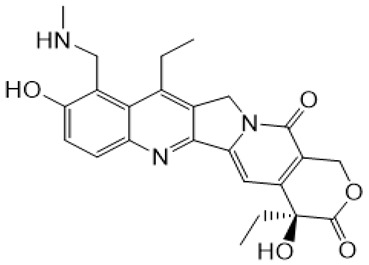 **NMe** **C_24_H_25_N_3_O_5_**	436.1867	436.1872 (1.2)	361.1545; 376.1071; 405.1438 (SN-38QM)
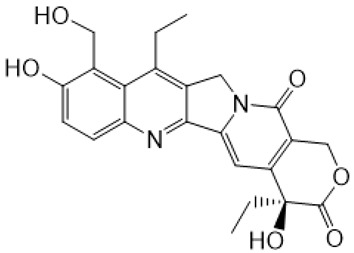 **9-CH_2_OH** **C_23_H_22_N_2_O_6_**	423.1556	423.1551 (0.0)	305.1291; 333.1595 361.1546; 405.1446 (SN-38QM)
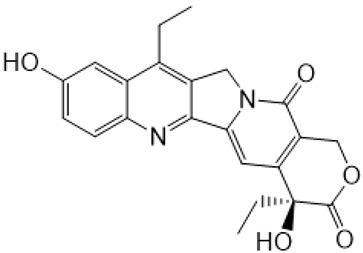 **SN-38** **C_22_H_20_N_2_O_5_**	393.1450	393.1445 (0.0)	212.0945; 249.1024; 264.1256; 293.1283; 321.1599; 349.1546
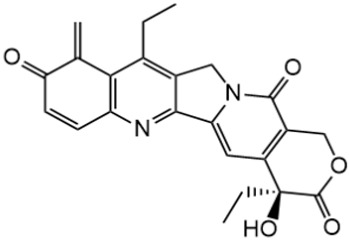 SN-38QM (quinone methide intermediate) * C_23_H_20_N_2_O_5_	405.1435	405.1443 (−0.4)	304.1200; 333.1607; 361.1546; 376.1086
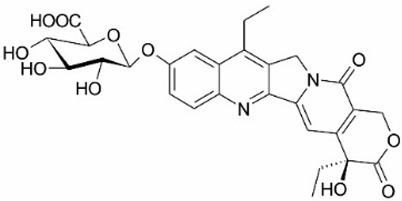 **SN-38G-β (β-D-glucuronide)** 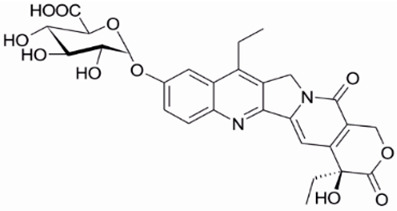 **SN-38G-α (α-D-glucuronide)** **C_28_H_28_N_2_O_11_**	569.1771	569.1756 (−1.8)	349.1537; 393.1446
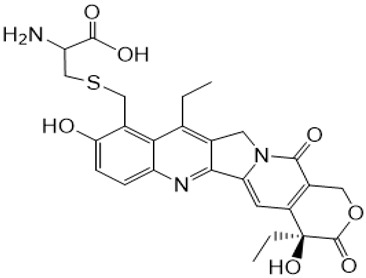 **9-CH_2_-S-Cys** **C_26_H_27_N_3_O_7_S**	526.1642	526.1642 (−0.1)	122.0268; 304.1200; 361.1546; 405.1445
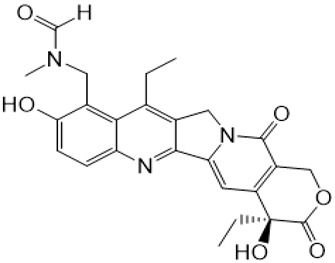 **NMe-formyl + NMe-formyl-D1** **C_25_H_25_N_3_O_6_; C_25_H_24_D_1_N_3_O_6_**	464.1816 (trace)	464.1816 (−0.1)	361.1563; 405.1449; 421.1429
	465.1889 (the most abundant monodeuterated)	465.1878 (−0.1)	361.1518; 405.1441; 422.1451

* it was found to be both a product ion in MS/MS spectrum and the product of in-source fragmentation found in MS spectrum.

**Table 2:** Structures of IR, metabolites, or photodegradation products detected and identified by HRMS and MS/MS fragmentation experiments (LCMS9050-Q-TOF system) in extracted biological samples.

Structure, Abbreviation and Formula	HRMS for [M + H]^+^ Calculated	HRMS Found (Error [ppm])	MS/MS Product Ions Masses
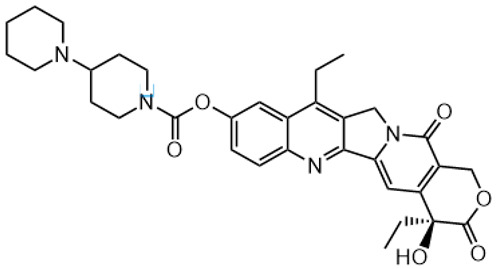 **IR** **C_33_H_38_N_4_O_6_**	587.2864	587.2871 (1.1)	124.1119; 167.1178; 195.1491; 331.1445; 458.2076; 502.1975; 543.2968
**SN-38** **C_22_H_20_N_2_O_5_**	(see Table 1)		
**SN-38G-β and SN-38G-α** **C_28_H_28_N_2_O_11_**	(see Table 1)		
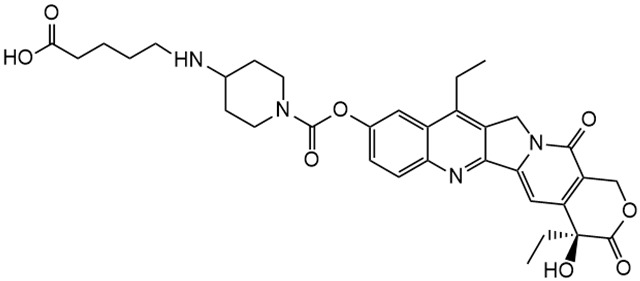 **APC** **C_33_H_38_N_4_O_8_**	619.2762	619.2764 (0.2)	227.1393; 393.1443
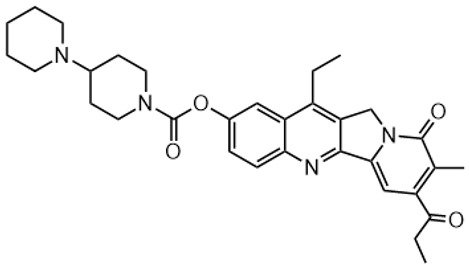 **PDP5** [31] **C_32_H_38_N_4_O_4_**	543.2966	543.2956 (−1.9)	ND *
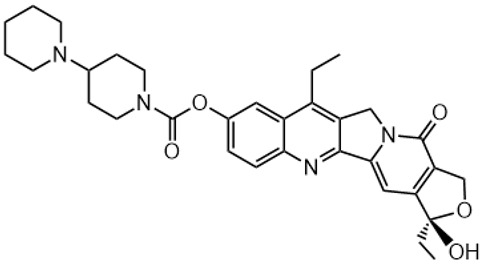 **PDP2** [31] **C_32_H_38_N_4_O_5_**	559.2915	559.2915 (0.2)	124.1117; 167.1179; 195.1491; 318.1000; 347.1383; 456.1919; 474.2021
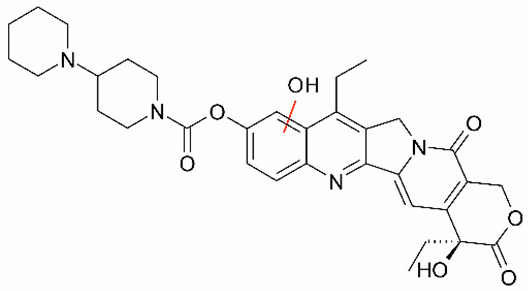 **IR-OH** **C_33_H_38_N_4_O_7_** –OH group attached to polycyclic SN-38 core with unassigned locant, Rt 14.3 min	603.2813	603.2820 (1.1)	124.1117; 167.1182; 195.1488; 518.1945; 541.2819; 585.2718
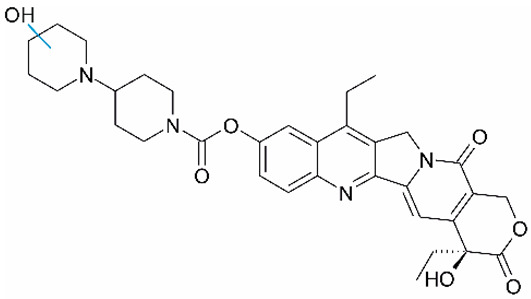 **IR-OH** **C_33_H_38_N_4_O_7_** –OH group attached to bipiperidine moiety with unassigned locant; Rt 15.3 min	603.2813	603.2827 (2.2)	458.2125; 502.1993
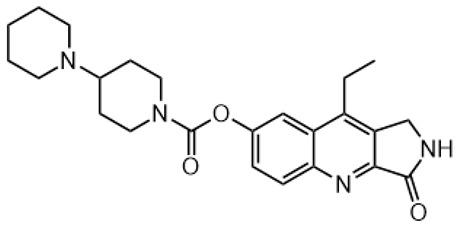 **IR-∆E** **C_24_H_30_N_4_O_3_**	423.2391	423.2390 (−0.3)	124.1187; 167.1174; 195.1487; 211.0848; 338.1498

* Not determined, tentative identification due to the absence of product ions.

**Table 3:** Mean EIC area peak for detected metabolites in plasma in timepoints with mean ratios of SN-38G to sum of others (systemic glucuronidation index—**SGI**).

**Plasma, NMe**	**15′ F**	**30′ F**	**60′ F**	**15′ M**	**30′ M**	**60′ M**
NMe	694	264	62	83	307	0
9-CH_2_OH	129,635	28,660	4560	10,819	10,892	101
SN-38	249,822	76,238	20,995	56,626	126,914	9321
SN-38G	48,362	34,245	21,593	6027	11,626	5868
9-CH_2_-S-Cys	1139	1087	69	525	322	0
**SGI**	**0.13**	**0.31**	**0.93**	**0.11**	**0.10**	**0.88**
Std. Dev.	0.04	0.09	0.32	0.07	0.08	0.45
**Plasma, IR**	**15′ F**	**30′ F**	**60′ F**	**15′ M**	**30′ M**	**60′ M**
Irinotecan	876,157	1,341,356	1,012,773	2,977,582	1,063,762	684,837
SN-38	69,712	69,843	112,091	494,868	19,203	49,170
SN-38G	45,392	10,052	7454	78,060	5579	7782
**SGI**	**0.04**	**0.01**	**0.01**	**0.02**	**0.01**	**0.01**
Std. Dev.	NA	NA	NA	NA	0.14	NA

F—female; M—male; NA—not applicable (*n* = 1).

**Figure A2:**
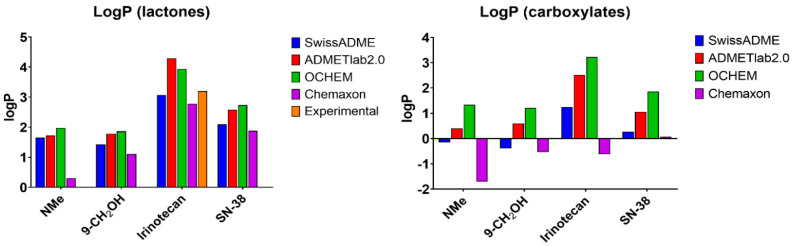
Comparison of logP values for lactones and carboxylates predicted with four chemoinformatic tools: SwissADME, ADMETlab2.0, OCHEM, and Chemaxon and experimental values (if applicable).

## Data Availability

The raw data will be made available by the corresponding authors on request.

## Abbreviations

The following abbreviations are used in this manuscript:

ADME	Absorption, Distribution, Metabolism, and Excretion of drug characteristics
ESI	Electrospray Ionization
FDR	False Discovery Rate
GSH	Glutathione
IR	Irinotecan
LCMS	Liquid Chromatography coupled with Mass Spectrometry
NAC	N-acetylcysteine
NMe	9-*N*-methylaminomethyl derivative of SN-38
PDP2/PDP5	Photodegradation products (of IR) no 2 or 5
SGI	Systemic Glucuronidation Index
TOCSY	Total Correlation Spectroscopy
TPT	Topotecan

## Appendix A

### Appendix A.1. Choice of Drug Administration Route

We have used the extravascular route of API administration because it secures high metabolite concentration after relatively short time after injection, while the intravascular route secures high plasma concentration of metabolites just after injection and faster elimination after distribution.

Extravascular (including intraperitoneal, *i.p.*) administration involves absorption to circulatory system during initial phase of time course of plasmatic drug concentration. Tillement et al. [52] presented that for most drugs and for the same dose, the intraperitoneal route drug level reaches 70% of the intravenous drug level in the circulatory system in humans based on the AUC parameter (Figure A1), while Guichard et al. [53] proved that plasmatic AUC values of IR and SN-38 are equivalent regarding *i.p*. and *i.v.* administration in mice bearing P388 leukaemia ascites with the same t_1/2_ for SN-38 and SN-38G and not significantly altered C_max_ and t_1/2_ for IR. Moreover, authors proved *i.p.* administration of IR to mice bearing C26 colon cancer is more efficient and less toxic than *i.v.* administration with toxic deaths at doses of 400 mg/kg and 800 mg/kg, *i.v.* and *i.p.* given, respectively.

### Appendix A.2. Chemometric Methods for Predicting ADME and Toxicity

We have used three chemoinformatic tools, Chemaxon [54], ALOGPS (OCHEM) [55], and ADMET-lab2.0 [56], to predict and compare some physicochemical parameters of IR, SN-38, NMe, and metabolite 9-CH_2_OH (Table A1). Similarly, we have predicted ADMET with two online predictor platforms: ADMETlab2.0 and SwissADME (Table A2). AdmetSAR, OCHEM, and Chemaxon predictions are commonly implemented in the DrugBank repository in complement to experimental values.

**Table A1:** Comparison of physicochemical properties for IR (CPT-11), SN-38, NMe, and 9-CH_2_OH in lactone and carboxylate form.

Property	Experimental/ Predicted	Value for IR	Value for SN-38	Value for NMe	Value for 9-CH_2_OH
Molecular Weight [g/mol]	Exact	586.28 ^a^	392.13 ^a^	435.18 ^a^	422.43 ^a^
		603.28 ^b^	409.14 ^b^	452.18 ^b^	439.15 ^b^
Melting Point [°C]	Exp	222–223	217	no data	no data
Water Solubility	Exp	Soluble; pH-dep.	Poor; pH-dep.	Soluble; pH-dep.	Soluble; pH-dep.
logP	Exp	3.20	no data	no data	no data
logP	Pred	3.94 ^a^	2.73 ^a^	1.98 ^a^	1.86 ^a^
		3.22 ^b^	1.86 ^b^	1.34 ^b^	1.21 ^b^
logP	Pred	2.78 ^a^	1.88 ^a^	0.30 ^a^	1.11 ^a^
		−0.62 ^b^	0.07 ^b^	−1.71 ^b^	−0.54 ^b^
logP	Pred	4.29 ^a^	2.58 ^a^	1.72 ^a^	1.78 ^a^
		2.52 ^b^	1.06 ^b^	0.41 ^b^	0.60 ^b^
logS	Pred	−3.70 ^a^	−3.13 ^a^	−3.48 ^a^	−3.07 ^a^
		−3.77 ^b^	−2.90 ^b^	−3.26 ^b^	−3.08 ^b^
logS	Pred	−5.54 ^a^	−4.32 ^a^	−3.66 ^a^	−3.77 ^a^
		−4.55 ^b^	−3.38 ^b^	−2.71 ^b^	−2.82 ^b^
logS	Pred	−5.03 ^a^	−4.25 ^a^	−3.78 ^a^	−4.00 ^a^
		−4.16 ^b^	−3.59 ^b^	−3.43 ^b^	−3.54 ^b^
pKa (Strongest Acidic)	Pred	11.71 ^a^	9.66 ^a^	10.48 ^a^	9.59 ^a^
		3.04 ^b^	3.36 ^b^	3.18 ^b^	3.29 ^b^
pKa (Strongest Basic)	Pred	9.47 ^a^	4.32 ^a^	7.85 ^a^	3.99 ^a^
		9.17 ^b^	4.38 ^b^	10.51 ^b^	4.09 ^b^
pI	Pred	10.44 ^a^	6.99 ^a^	9.15 ^a^	6.79 ^a^
		6.71 ^b^	3.77 ^b^	5.63 ^b^	3.64 ^b^
Physiological Charge *	Pred	1 ^a^	0 ^a^	1 ^a^	0 ^a^
		0 ^b^	0 ^b^	0 ^b^	0 ^b^
Formal Charge	Pred	0 ^a^	0 ^a^	0 ^a^	0 ^a^
		−1 ^b^	−1 ^b^	−1 ^b^	−1 ^b^
HA Count	Pred	6 ^a^	5 ^a^	6 ^a^	6 ^a^
		8 ^b^	7 ^b^	8 ^b^	8 ^b^
HA Count	Pred	10 ^a^	7 ^a^	8 ^a^	8 ^a^
		11 ^b^	8 ^b^	9 ^b^	9 ^b^
HD Count	Pred	1 ^a^	2 ^a^	3 ^a^	3 ^a^
		3 ^b^	4 ^b^	5 ^b^	5 ^b^
HD Count	Pred	1 ^a^	2 ^a^	3 ^a^	3 ^a^
		2 ^b^	2 ^b^	4 ^b^	4 ^b^
TPSA [Å2]	Pred	112.51 ^a^	99.96 ^a^	111.99 ^a^	120.19 ^a^
		143.74 ^b^	131.19 ^b^	143.22 ^b^	151.42 ^b^
TPSA [Å2]	Pred	114.20 ^a^	101.65 ^a^	113.68 ^a^	121.88 ^a^
		148.26 ^b^	134.20 ^b^	147.74 ^b^	155.94 ^b^
Bioavailability (F) **	Pred	yes	yes	yes	yes
Rule of Five **	Pred	no	yes	yes	yes

^a^ denotes properties for lactone form, ^b^ denotes properties for carboxylate; HA—H-bond acceptor; HD—H-bond donor; TPSA—topological polar surface area; * Chemaxon does not take carboxylates as anions; it transfers structures to protonated forms with no charge; ** is the same for both forms.

**Table A2:** Predicted ADMET parameters for two drugs: NMe and IR and two metabolites: 9-CH_2_OH and SN-38 in lactone (left) and carboxylate (right) forms.

Property	NMe	9-CH_2_OH	IR	SN-38	NMe	9-CH_2_OH	IR	SN-38
Drug/prodrug	drug	active metabolite	prodrug	drug	drug	active metabolite	prodrug	drug
Pgp-inh	no	no	no	no	no	no	no	no
Pgp-sub	yes	yes	yes	yes	yes	yes	yes	yes
HIA	h	h	h	h	h	h	h	h
F(30%)	0.125	0.055	0.277	0.006	0.005	0.167	0.037	0.006
Caco2 permeability	p	g	p	g	p	p	p	g
MDCK permeability	m	m	h	h	m	m	h	h
BBB penetration	m	e	e	e	e	e	e	e
PPB	e	p	e	p	e	p	e	p
VD [L/kg]	2.02	1.04	2.94	0.95	1.98	0.97	2.03	0.85
Frac unbound	16.9%	6.3%	25.7%	4.9%	25.6%	8.9%	31.8%	8.1%
CYP1A2-substrate	yes	yes	no	yes	yes	no	no	No
CYP2C1-substrate	no	no	yes	no	no	no	yes	no
CYP2C9-substrate	no	no	no	no	no	no	no	no
CYP2D6-substrate	no	no	no	no	no	no	no	no
CYP3A4-substrate	no	no	yes	no	no	no	yes	no
CL	e	e	p	e	p	p	p	p
T1/2	e	p	e	m	p	p	e	p
hERG blocker	m	e	p	e	e	e	m	e
H-HT	p	m	p	m	m	m	p	p
DILI	e	g	m	g	e	g	g	g
Ames toxicity	p	m	p	p	g	g	h	g
Rat Oral Acute tox.	e	p	p	p	e	e	p	e
Carcinogenicity	e	e	e	e	e	e	e	e
Eye corrosion	e	e	e	e	e	e	e	e
Eye irritation	e	e	e	e	e	e	e	e
Respiratory tox.	e	e	m	e	m	e	m	e
QED	g	g	m	g	m	m	m	g
Lipinski rule	a	a	a	a	a	a	r	a

e—excellent, h—high, g—good, m—medium, p—poor, a—accepted, r—rejected.

LogP values have been predicted with 4 online platforms and compared in Figure A2.

Regarding physicochemical properties such as logP and solubility, four different tools were tested: ADMETlab, SwissADME, OCHEM, and Chemaxon. OCHEM calculates logP with the ALOGPS program based on SMILES code and SwissADME as the arithmetic average of the values predicted by five methods (XLOGP3, WLOGP, MLOGP, SILICOS-IT, and iLOGP). Values of logP obtained from these methods for IR varies from 2.55 to 4.95, and for NMe, they are more consistent. WLOGP algorithm showed for IR the closest to experimental (3.2) logP score of 3.1. For this reason, we decided to employ here WLOGP-derived values. Among tested 18 websites by Dulsat et al. tools [57], OCHEM showed the best fit for logP. In terms of pK_a_ obtained with Chemaxon (Table A1), NMe has a lower pK_a_ value than for IR (10.48 and 11.71, respectively), with pK_b_ (for strongest basic form) of 7.85 vs. 9.47 for IR. This indicates higher acidic character for NMe. SN-38 has an even lower pKa of 9.66 (higher acidity). IR is a stronger base in this evaluation (~11). Isoelectric points (pI) also vary from 6.99 for NMe to 10.44 for SN-38. Solubility predictions (logS) were not considered.

**The complexity of absorption and solubility predictions in camptothecins case.**

Camptothecins illustrate the inherent limitations of current in silico predictions of aqueous solubility and drug oral absorption. Such predictions are often difficult to interpret and may lack practical relevance or be misleading. Aqueous solubility and oral bioavailability remain major causes of drug development failure. Notably, a comparative assessment of solubility (logS) predictions for tyrosine kinase inhibitors across many tested online platforms showed that none achieved consistently accurate results [6].

Under physiological conditions, camptothecins exist in a pH-dependent equilibrium between lactone and carboxylate forms. The carboxylate form, due to its negative charge and high polarity, is markedly more soluble, whereas the lactone form is pharmacologically active but poorly soluble. The equilibrium ratio varies among derivatives, and dissolution of the lactone form in aqueous buffers at pH > 5 inevitably leads to gradual conversion to the carboxylate form, further complicating solubility assessment.

Several predictive algorithms fail to account for the influence of the isoelectric point (pI), despite its critical role in solubility. When pI approaches the pH of the solution, compounds are predominantly uncharged, resulting in minimal aqueous solubility. For example, the OCHEM algorithm neglects pI effects and predicts logS mainly based on polarity descriptors such as logP and polar surface area. Solubility predictions are also often disproportionately influenced by logP values. These simplifying assumptions introduce cumulative errors, leading to high variability and reduced credibility of predictions.

An optimal predictive approach would allow user-defined physicochemical inputs and explicitly consider all relevant chemical species at a given pH. Even then, reliable prediction remains challenging, as each ionization state must be evaluated independently. For instance, Chemicalize predicts 7 lactone and 12 carboxylate species of irinotecan coexisting in aqueous buffers, underscoring complexity.

For similar reasons, absorption predictions would be here unreliable. Lactone and carboxylate forms differ in their affinities toward membrane proteins and transporters, engaging distinct mechanisms such as ABC transporter-mediated efflux, active transport, passive diffusion, and facilitated diffusion. Consequently, accurate prediction of pharmacokinetic parameters (C_max, t_max) is often unattainable, even for small molecules with well-defined physicochemical properties, necessitating empirical evaluation.

Despite these limitations, predicted ADME parameters remain useful as qualitative guidance and are widely incorporated into pharmaceutical databases, including DrugBank. However, both lactone and carboxylate forms must be explicitly considered. When both species act as substrates for the same enzyme, competitive binding may occur, potentially increasing and prolonging plasma exposure (AUC) of co-administered drugs. In the case of irinotecan, the presence of carboxylates may enhance clearance of the active lactone, contributing to severe toxicity, including organ failure or death. High predictive accuracy of ADMETlab and SwissADME for multiple physicochemical and ADME parameters has been reported [6], demonstrating statistically significant agreement with experimental data for tyrosine kinase inhibitors, as confirmed by pairwise *t*-test analysis.

**Absorption**

Based on predictions, IR has the most favourable expected oral absorption with a probability of bioavailability < 30% (F30%) close to 1. All four compounds are predicted to be PGP-substrates, but not inhibitors. This parameter validation was not assessed in predictors comparison [57]; however, absorption is a complex process and has to be experimentally determined. So far, absorption predictions are rather irrelevant directions. Human intestinal absorption (HIA) is well predicted by SwissADME (*p* > 0.05 on pairwise *t*-test vs. experimental value, not significant), and here, SwissADME and ADMETlab complied for high HIA. HIA, F30% probability, and PGP-substrate together suggest good oral bioavailability for both IR and NMe. IR indeed is administered orally as one of the therapeutic options (Camptosar^®^)

**Distribution**

NMe is predicted to partly be able to penetrate blood–brain barrier (BBB), while 9-CH_2_OH, IR and SN-38 are unable. This is related to the higher polar character of NMe, due to protonable (susceptible to protonation) aminoalkyl NHMe substituent and, in a result, lower logP. NMe and IR showed similarly good plasma protein binding (PPB) parameters, and in plasma, 17 and 26% of NMe and IR, respectively, is assumed to be unbound (serum albumins) compared to only 5% for 9-CH_2_OH and SN-38. Optimal PPB is < 90% (unbound > 10%). Drugs with high PBB (95–100%) in serum can have a lower therapeutic index. Dulsat et al. [57] reported ADMETlab was more accurate for PPB prediction than ADMETSAR

Regarding **excretion**, the predictor ADMETlab showed for NMe the greatest clearance, CL, which is related to low requirement in terms of frequency of dosing a drug. IR had the lowest predicted CL. Drug clearance by metabolism and elimination dictates maintenance dosing concentrations. The liver and kidneys are responsible for metabolism and elimination, respectively. Blood flow and uptake in these organs dictate the extent of clearance. Clearance is generally a well-predicted parameter by ADMETlab [57].

Regarding **metabolism** by intrahepatic cytochrome P450 CYP isoenzymes, SN-38 and 9-CH_2_OH are not substrates for CYP enzymes. NMe is predicted not to be a CYP3A4 substrate. Drug inhibitory activities toward CYP enzymes were not predicted here. IR is metabolized by CYP3A4 and CYP3A5 to some inactive metabolites, which complies experimentally and computationally. NMe, contrary to IR, is predicted to be a CYP1A2 inhibitor and substrate, and this would mean that co-administration of caffeine, propranolol, clozapine, flutamide, and some chemotherapeutics such as imatinib could affect its absorption and therapeutic efficacy. In comparison, ADMETlab correctly predicted CYP3A4 substrates with 75% accuracy, while AdmetSAR with 100% accuracy. For CYP enzymes inhibiting activity, evaluation could not be performed, and this was not predicted.

Regarding **toxicity**, IR presented the highest probability for cardiac toxicity (hERG), highest for hepatotoxicity (H-HT), rat oral acute toxicity (ROA), and medium for respiratory toxicity, and indeed, IR in clinics in monotherapy shows the mentioned adverse effects. Comparison showed notably lower expected general toxicity for NMe: (a) medium cardiac toxicity probability, (b) high hepatotoxicity, (c) low respiratory toxicity, and (d) lower rat oral acute toxicity. 9-CH_2_OH showed low cardiac toxicity probability, low carcinogenicity, and the lowest respiratory toxicity. Respiratory toxicity has become the main cause of drug withdrawal. However, there is some probability of medium hepatotoxicity for 9-CH_2_OH. All four showed high mutagenicity (Ames) and low carcinogenicity, and among these, NMe showed the lowest carcinogenicity. None of the four camptothecins is expected to irritate skin or eyes. These predictions should be, however, taken with caution and rather as a complement to experimental data, as it can be misguiding as a stand-alone method.

### Appendix A.3. Supplementary Information

**Table A3:** Linearity with concentration ranges for IR and SN-38 obtained with LCMS9050-Q-TOF method used for kidney or liver extracts.

Parameter	IR				SN-38		
	Kidney M	Kidney F	Liver (M/F)		Kidney M	Kidney F	Liver (M/F)
Conc. range [ng/uL]	1–15	0.5–20	1–50	0.2–10	0.04–2	0.5–5	0.1–0.5
R^2^	0.9976	0.9982	0.9975	0.9964	0.9999	0.9915	0.9998

At least four (up to six) concentration data points were used for linear regression. Lower SN-38 concentrations were prepared for calibration standards, as we observed low amounts of SN-38 in irinotecan groups.

**Table A4:** Concentrations [ng/µL] for IR and SN-38 obtained with LCMS9050-Q-TOF calibration curve method.

Matrix	Plasma		Liver		Kidney		Lungs		Matrix	Stool
Group	IR	SN-38	IR	SN-38	IR	SN-38	IR	SN-38	Grroup	SN-38
IR 15′ F	1.55	0.08	5.74	0.12	4.22	btr	2.40	btr	NMe 15′ F	btw
	nd	nd	4.85	0.10	4.34	0.56	1.68	btr		0.16
	nd	nd	nd	nd	4.16	btr	2.29	btr		0.20
IR 30′ F	0.86	0.03	5.72	0.12	3.61	btr	0.71	btr	NMe 30′ F	nd
	nd	nd	5.37	0.10	5.72	btr	1.57	btr		nd
	nd	nd	3.98	btr	3.58	btr	nd	nd		nd
IR 60′ F	btr	0.04	3.16	btr	nd	nd	0.59	btr	NMe 60′ F	0.89
	nd	nd	4.26	0.10	4.12	btr	0.29	btr		0.61
	nd	nd	4.33	btr	3.10	btr	0.65	btr		0.92
IR 15′ M	1.90	0.02	8.51	0.11	4.41	0.07	0.74	btr	NMe 15′ M	nd
	nd	nd	11.39	0.10	4.16	btr	1.29	btr		nd
	nd	nd	5.90	btr	3.94	0.05	1.23	btr		nd
IR 30′ M	1.26	0.02	10.25	0.11	4.48	0.04	1.06	btr	NMe 30′ M	nd
	btr	btr	3.31	btr	2.75	btr	1.20	btr		nd
	nd	nd	nd	nd	nd	nd	1.81	btr		nd
IR 60′ M	btr	0.03	1.73	btr	1.33	0.05	0.50	btr	NMe 60′ M	nd
	nd	nd	5.10	btr	2.38	0.04	0.69	btr		nd
	nd	nd	8.11	0.11	3.32	0.06	1.02	btr		nd

**Table A5:** Concentrations [ng/mg tissue] for IR and SN-38.

Matrix	Liver		Kidney		Lungs
Group	IR	SN-38	IR	SN-38	IR
IR 15′ F	49.2	1.0	23.4	0.3	27.2
	28.0	0.5	26.8	3.5	23.4
	nd	nd	22.4	0.1	19.8
IR 30′ F	85.8	1.8	21.9	0.1	<LOQ
	32.2	0.5	33.0	0.1	16.4
	29.9	0.5	20.1	nd	nd
IR 60′ F	27.1	0.3	nd	nd	<LOQ
	25.6	0.5	19.5	0.1	<LOQ
	39.4	0.5	18.4	<LOQ	<LOQ
IR 15′ M	19.3	0.4	23.2	0.4	10.0
	23.7	0.3	25.7	0.2	6.4
	12.7	0.2	22.3	0.3	7.5
IR 30′ M	23.9	0.5	23.2	0.2	11.0
	11.9	0.3	16.2	0.2	16.0
	nd	nd	nd	nd	14.1
IR 60′ M	<LOQ	<LOQ	7.0	0.3	6.0
	9.7	0.3	12.4	0.2	6.7
	15.4	0.4	18.0	0.3	9.8

**Table A6:** Concentrations [ng/µL] (range in case of triplicate) of SN-38 in IR and NMe groups.

**Matrix, Group**	**15′ F**	**30′ F**	**60′ F**
plasma, IR	0.02–0.08	0.01–0.03	0.01–0.04
plasma, NMe	0.15	0.11	0.07
stool, IR	0.02–0.04	0.03–0.04	0.02–0.03
stool, NMe	0.16–0.20	0.29	0.61–0.92
**Matrix, Group**	**15’ M**	**30′ M**	**60’ M**
kidney, IR	nd	0.04	nd
kidney, NMe	nd	0.28	nd

**Table A7:** EIC peak areas for NMe and metabolites in plasma extract sample no 12F before and after 6 months storage at −80 °C (LCMS method).

Plasma Sample 12F	NMe	SN-38G	9-CH_2_-S-Cys	Alk-9	SN-38
start	112.00	24,803.41	0.00	8 543.11	18,455.00
after 6 months	0.00	24,192.93	0.00	781.05	18,242.11
